# Volatiles emitted by the entomopathogenic fungus *Beauveria bassiana* elicit growth and defense in sorghum plants

**DOI:** 10.3389/ffunb.2025.1725103

**Published:** 2026-01-19

**Authors:** Sandra Goretti Adame-Garnica, Arturo Ramírez-Ordorica, Vicente Montejano-Ramírez, Elda Castro-Mercado, Patricia Ríos-Chávez, Ernesto García-Pineda, Eduardo Valencia-Cantero, Lourdes Macías-Rodríguez

**Affiliations:** 1 Instituto de Investigaciones Químico Biológicas, Universidad Michoacana de San Nicolás de Hidalgo, Ciudad Universitaria, Morelia, Michoacán, Mexico; 2 Facultad de Biología, Universidad Michoacana de San Nicolás de Hidalgo, Ciudad Universitaria, Morelia, Michoacán, Mexico

**Keywords:** fungal volatiles, fungi and plant interaction, phenolic compounds, phytohormones, reactive oxygen species

## Abstract

**Background:**

*Beauveria bassiana* is an entomopathogenic fungus that can establish an intimate endophytic relationship with plants. Otherwise, microbial volatile organic compounds (VOCs) are important chemicals for plant recognition and interactions. Therefore, this study provides novel evidence of the biochemical and physiological responses of plants to VOCs emitted by *B. bassiana* and 3-methylbutanol (3MB) as the most abundant compound emitted by the fungus.

**Methods:**

Sorghum plants were exposed to the standard 3MB and VOCs emitted by the fungal strains AS5 and AI2 of *B. bassiana* isolated from soil and a mycosed insect cadaver, respectively. The accumulation of reactive oxygen species (ROS) such as superoxide anion (O_2_^•^¯) and H_2_O_2_; quantification of phytohormones such as salicylic acid (SA), jasmonic acid (JA), and indole-3-acetic acid (IAA) and phenolic compounds in leaves (4-coumaric acid and flavonoids); and the expression of genes *SbPR-1* and *SbCOI1* related to the activation of SA- and JA-signaling defense pathways, respectively, were analyzed.

**Results and discussion:**

VOCs emitted by *B. bassiana* and 3MB stimulate plant growth, likely by triggering the production of ROS and IAA. Furthermore, these fungal compounds increased the expression levels of *SbPR-1* and *SbCOI1* at 2 d and *SbCOI1* at 7 d. Consistently, an increase in the content of SA, JA, and phenolic compounds was observed in the inoculated plants.

**Conclusion:**

VOCs emitted by *B. bassiana* and 3-MB promote sorghum growth and activate adaptive defense traits. Moreover, VOCs from AS5 triggered a stronger biochemical response in plants than VOCs emitted by AI2. These results suggested that the response of the plant was strain-specific. Finally, 3MB is a fungal compound that may stimulate plant growth and defense.

## 1 Introduction

*Beauveria bassiana* (Hypocreales: Clavicipitaceae) is an entomopathogenic fungus that infects and kills insects at any stage of development (Hintènou et al., 2023; Gielen et al., 2024). Furthermore, *B. bassiana* can colonize other trophic niches, such as soil and plants, adding value to the fungus as a bioinoculant in agroecosystems (Raya-Díaz et al., 2017; Ortiz-Urquiza, 2021; Quesada-Moraga et al., 2023; Sui et al., 2023). The beneficial effects of *B. bassiana* as an endosymbiont of plants include: i) nutrient and water uptake from soil, ii) antagonistic activity against phytopathogens, iii) activation of the antioxidant system, iv) induction of systemic resistance against herbivore and phytopathogen attackers, and v) attraction of the natural enemies of herbivores (Raya-Díaz et al., 2017; Rondot and Reineke, 2019; González-Mas et al., 2021; Shaalan et al., 2021; Gupta et al., 2022; Homayoonzadeh et al., 2022; Proietti et al., 2023; Liu et al., 2025; Yousef-Yousef et al., 2025). Despite these fungal attributes, little is known about the effect of chemicals that are released by *B. bassiana* that mediate its “communication” with the plants to form beneficial associations.

Soil microbes emit volatile organic compounds (VOCs) as chemical cues to interact with plants and elicit numerous physiological and biochemical processes (Ryu et al., 2003; Sánchez-López et al., 2016; Moisan et al., 2020; El Jaddaoui et al., 2023; Montejano-Ramírez et al., 2024). The type and magnitude of plant responses vary according to microbial VOC signatures and environmental contexts (Gutiérrez-Luna et al., 2010; Fincheira et al., 2021). Similarly, pure compounds such as 2,3-butanediol, acetoin, dimethyl disulfide, C16-dimethylamine, 6-pentyl-2*H*-pyran-6-one, and 3-methylbutanal identified from *Bacillus subtilis* GB03, *B. methylotrophicus* M4-96; *Pseudomonas stutzeri* E25, *Arthrobacter agilis* UMCV2, *Trichoderma atroviride* IMI206040, and *Cladosporium halotolerans* NGPF1, respectively, have been assayed and validated as plant growth promoters, elicitors of plant defense or in the suppression of pathogens in different plant species (Ryu et al., 2003; Garnica-Vergara et al., 2016; Raya-González et al., 2017; Rojas-Solís et al., 2018; Vicente-Hernández et al., 2019; Jiang et al., 2021). Nevertheless, the substance’s volatility, experimental conditions, and exposure time may influence the effectiveness of the compounds.

The effects of VOCs emitted by entomopathogenic fungi on plants have not yet been addressed. However, they can emit a wide range of chemical compounds, including fatty acids, terpenes, aldehydes, ketones, and alcohols (Bojke et al., 2018), which could participate in “communication” with plants. Ramírez-Ordorica et al. (2022, 2024) analyzed the VOC profiles of different strains of *B. bassiana*. The strains isolated from mycosed insect cadavers were more virulent against the fall armyworm (*Spodoptera frugiperda*) than those isolated from the soil. VOC analysis revealed differences among the fungal strains. However, they all emitted 3-methylbutanol (3MB), with abundances ranging from 54 to 72%. Thus, 3MB may be a biomarker compound attributable to the presence of *B. bassiana*. Interestingly, the most virulent strain AI2 had the lowest emission of 3MB, whereas strain AS5 had the highest. Bioassays performed with *S. frugiperda* showed that fungal VOCs and 3MB modulated the oviposition and food choice preference in larvae fed with sorghum plants (*Sorghum bicolor*). The chemical analysis performed by ultra-performance liquid chromatography and mass spectrometry (UPLC-MS) from leaves exposed to fungal VOCs revealed an increased abundance of 4-coumaric acid, a phenolic acid with a potent antioxidant activity in plants and an important role in plant defense. Hence, using strains AS5 and AI2, we aimed to answer the following questions: What are the biochemical and physiological responses of plants to VOCs emitted by the entomopathogenic fungus *B. bassiana*? Furthermore, do differences in the emission of fungal VOC induce strain-specific responses in plants?

## 2 Materials and methods

### 2.1 Sorghum growing conditions

Seeds (*Sorghum bicolor* cv. Fortuna) were vigorously shaken in 15% ethanol for 5 min. Then, the seeds were washed with sterile deionized water and immersed in 10% commercial bleach for 5 min. Finally, the seeds were rinsed five times with sterile deionized water. Seed germination was performed in a Petri dish using 10 mL of Hoagland medium, (10.2 g/L KNO_3_, 4.92 g/L, Ca(NO_3_)_2_ × 4H_2_O, 2.3 g/L NH_4_H_2_PO_4_; 4.9 g/L MgSO_4_ × 7H_2_O, 28.6 mg/L H_3_BO_3_, 18.1 mg/L MnCl_2_ × 2H_2_O, 0.8 mg/L CuSO_4_ × 5H_2_O, 2.2 mg/L ZnSO_4_ × 7H_2_O, 0.9 mg/L Na_2_MoO_4_ × 2H_2_O, 30 mg/L FeSO_4_, 10 g/L agar plant TC). The Petri dish was placed in a plant growth chamber with a 16/8 h photoperiod (light/dark) at 22°C. Four days after germination, embryonic axes were carefully separated from the endosperm using a scalpel (Hernández-Calderón et al., 2018). The embryonic axes were disinfected with 5% commercial bleach, rinsed five times with sterile deionized water, and finally placed in 150 mL glass flasks containing 30 mL Hoagland medium. The seedlings were incubated for 8 d in a growth chamber under the same conditions as described above.

### 2.2 Interaction system of *S. bicolor* and *B. bassiana* via volatiles emission

**Figure 1:**
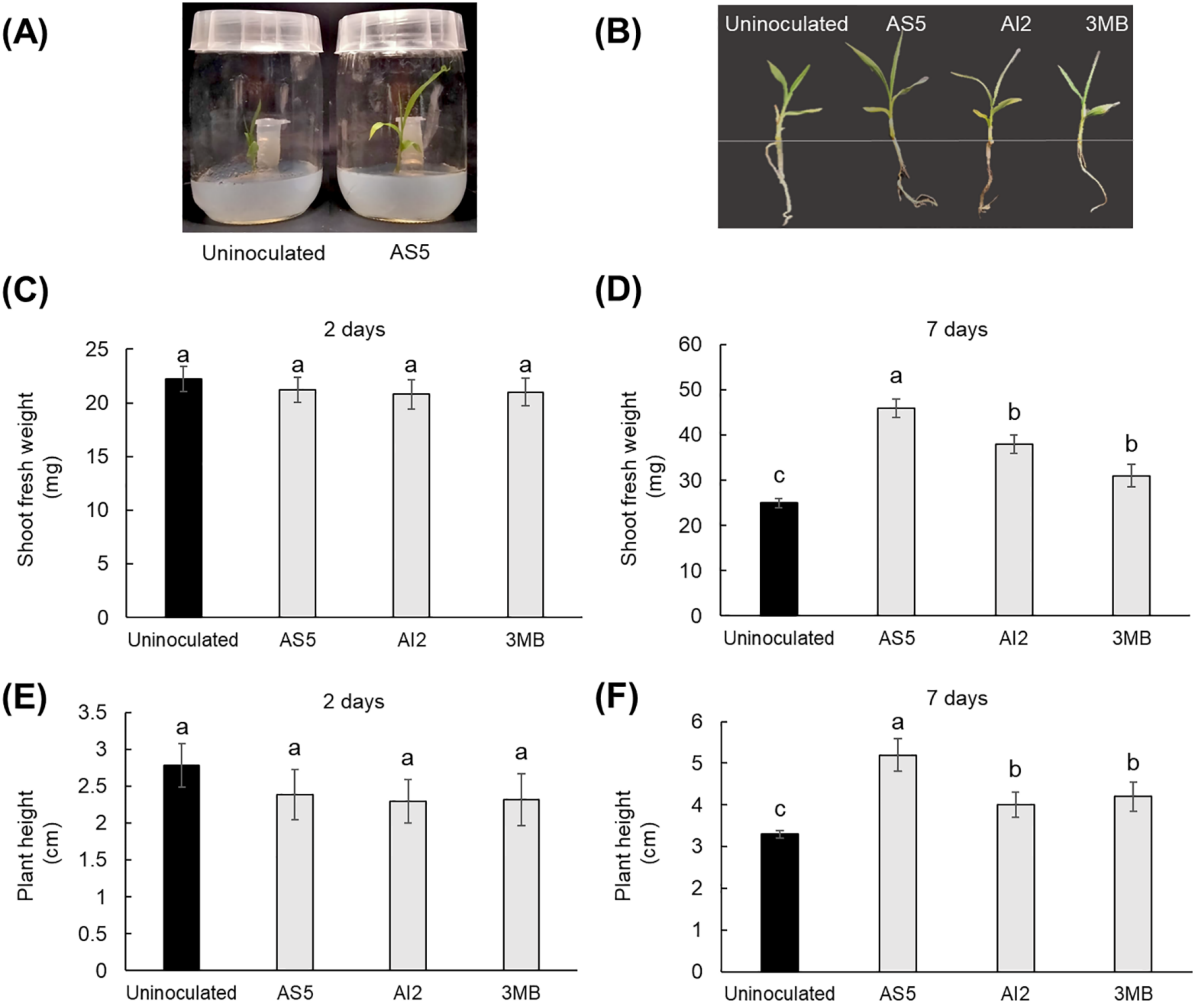
Effects of VOCs emitted by *B*. *bassiana* and 3-methylbutanol on the shoot growth of sorghum plants at 2 and 7d of the interaction. **(A)** Representative photograph of the interaction system in which the plant and the strain AS5 interacted via VOCs emission for 7 d. **(B)** Seedlings of 7-day-old control uninoculated plants and plants exposed to VOCs emitted by the strains AS5 and AI2 and the fusel alcohol. **(C–F)** Shoot fresh weight and plant height measured at 2 and 7 d after exposure with fungal VOCs. Bars represent the mean ± standard error values (n = 5). Different letters are used to indicate means that differ significantly by one-way ANOVA and Tukey’s *post-hoc* test (*p* ≤ 0.05).

Eight-day-old plants growing in 150 mL glass flasks were subjected to three different treatments. For treatments AS5 and AI2, a 2 mL Eppendorf tubes with 1 mL PDA medium were inoculated with 1 × 10^6^ spores of the AS5 or AI2 strains and inserted into the flask (**Figure 1A**). For the 3MB treatment, standard 3-methylbutanol (*>* 98.5%, Sigma-Aldrich^®^, catalog I9392) at 8.97 μM was placed in 2 mL Eppendorf tubes containing 1 mL PDA medium. The control treatment consisted of 2 mL Eppendorf tubes containing 1 mL PDA medium. Shoot fresh weight and plant height were recorded at 2 and 7 d after the interaction.

### 2.3 Histochemical staining to detect superoxide anion (O_2_^•^¯) in *S. bicolor* plants

The nitro blue tetrazolium (NBT) staining method was used to detect the *in situ* production of O_2_^•^¯ in leaves (Flohé and Ötting, 1984). This method is fast and sensitive and it is based on the reduction of the colorless NBT to a blue formazan precipitate by O_2_^•^¯. At 24 h after the fungal interaction, leaves and roots from each treatment were incubated in a 0.25% (w/v) NBT solution prepared in 10 mM KH_2_PO_4_, (pH 7.8) for 30 min at room temperature under dark conditions. The tissues were then distaining with boiling ethanol for 30 min. The tissues were then observed under a microscope (Nikon Eclipse E200); a blue color indicated the presence of superoxide anion.

### 2.4 Diaminobenzidine staining for *in vivo* detection of H_2_O_2_ in *S. bicolor* plants

To detect the production of H_2_O_2_ in plants after 24 h of fungal interaction, we used the DAB test (Thordal et al., 1997). DAB was oxidized by H_2_O_2_ to produce a dark brown polymerization product that required peroxidase activity. A DAB aqueous solution (1 mg/mL) was prepared at a pH of 3.0. The detached leaves and roots were immersed in a DAB solution and incubated at room temperature for 30 min, until a brown precipitate formed. Finally, plant tissues were washed with 96% ethanol. The stained tissues were observed under a microscope (Nikon Eclipse E200) for documentation.

### 2.5 Analysis of the expression of defense-related genes in sorghum plants

Gene expression was analyzed according to the methodology described by Hernández-Calderón et al. (2018). Gene specific primers pairs (**Supplementary Table S1**) were designed using the *S. bicolor* genome database (http://plantgdb.org/SbGDB/) to perform quantitative PCR (qPCR) of the defense genes *SbCOI1* (encoding the “protein coronatine-insensitive 1,” a marker of the jasmonic acid-inducible pathway) and *SbPR-1* (encoding the protein “pathogenesis related 1,” a marker of the salicylic acid-inducible pathway).

The plants under each experimental condition were frozen using liquid nitrogen. RNA was extracted with TRI reagent (Sigma-Aldrich^®^) and treated with 0.5 μL DNase I to remove DNA contamination. To assess the integrity of total RNA, electrophoresis was performed on a 1.2% agarose gel stained with GelRed (Biotium, USA). Final RNA concentration was estimated by using a NanoDrop spectrophotometer (Thermo Scientific, Rockford, IL, USA). cDNA was synthesized from 500 ng of the RNA template using the Super Script First-Strand Synthesis System (Life Technologies/Gibco-BRL, CA, USA). Quantitative PCR (qPCR) reactions (20 μL) were carried out using the iQ SYBR Green kit (Applied Biosystems) with 10 μL of master mix and 1 μL of gene-specific primers. The actin gene (ACT) was used to normalize the amount of *S. bicolor* RNA in each sample. Finally, gene expression was calculated using the mathematical formula: 2^−ΔΔCt^ (Livak and Schmittgen, 2001).

### 2.6 Analyses of phytohormones and 4-coumaric acid in leaves by gas chromatography and mass spectrometry

Salicylic acid (SA), jasmonic acid (JA), indole-3-acetic acid (IAA), and 4-coumaric acid analyses were analyzed by GC-MS. Leaf tissues were frozen and ground in liquid nitrogen. The ground tissues (200 mg) were placed in Eppendorf tubes and 500 μL isopropanol/H_2_O/HCl (2:1:0.002, v/v) were added for compound extraction. The samples were vortexed for 30 s and centrifuged at 11,500 rpm for 3 min. The supernatant from each sample was placed in a 5 mL reaction vial containing 300 μL dichloromethane and evaporated to complete dryness with gaseous nitrogen (purity ≥ 99.998%). The carboxyl group in each acidic molecule was methyl-esterified with 600 μL of acetyl chloride in methanol (500 μL/2 mL). The samples were sonicated for 30 min and heated in a thermoblock at 75°C for 1 h. After cooling, the derivatized samples were evaporated to dryness under a stream of gaseous nitrogen. Finally, dry samples were redissolved in 25 μL ethyl acetate, and 1 μL was injected into the injection port of a gas chromatograph (Agilent 7890B; Agilent, Foster City, CA, USA) equipped with an HP-5MS capillary column (30 m × 0.25 mm ID; film thickness 0.25 μm, Agilent), coupled with a mass spectrometer (5977A; Agilent) with Mass Hunter Workstation Software (Agilent Technologies, Santa Clara, CA, USA) for data acquisition and processing. Operating conditions were as follow: 1 mL min^−1^ helium as the carrier gas, the detector and injector temperatures were 300°C and 270°C, respectively. The column was held for 3 min at 150°C and programmed at 6°C min^−1^ to a final temperature of 278°C for 10 min. Selected-ion monitoring was used to analyze the methyl-esterified compounds. Therefore, pure standards were also methyl-esterified and used as references to obtain the retention times (min) and mass spectra. For SA, the mass-to-charge ratios (*m/z*) were 120 and 152 *m/z*, 1.906 min (**Supplementary Figure 1**); JA, 83 and 224 *m/z*, 4.19 min (**Supplementary Figure 2**); IAA, 130 and 189 *m/z*, 10.003 min (**Supplementary Figure 3**); and 4-coumaric acid, 147 and 178 *m/z*, 6.396 min (**Supplementary Figure 4**). The standards were used for quantification by correlating the injected concentration with the peak area of the eluted compound in the sample.

### 2.7 Determination of total phenolic compounds and total flavonoid content in sorghum plants

Leaves (20 mg) were ground with liquid nitrogen and macerated in the dark at room temperature with 5 mL of methanol for 48 h. The sample was then concentrated to a final volume of 1 mL using gaseous nitrogen. Total phenol content was spectrophotometrically determined using the Folin-Ciocalteau method, and gallic acid was used as a reference standard. A sample (20 µL) was mixed with 80 µL of deionized water and 500 µL 1N Folin-Ciocalteau reagent for 5 min. Later, 500 µL 7% Na_2_CO_3_ and 2.5 mL deionized water were added, and the mixture was left in the dark for 60 min. The absorbance was determined at 765 nm using an ultraviolet-visible (UV-VIS) spectrophotometer (VE-5100UV). For the reference curve, gallic acid was used at concentrations ranging from 0.05 mg/mL to 0.5 mg/mL; with the standard solutions prepared in the same manner as the samples. Total phenolic acid content was expressed as milligrams of gallic acid equivalent (GAE) per gram of fresh weight (FW) (Rodríguez-Valdovinos et al., 2021).

Flavonoid content was determined according to a method previously reported by Chang et al. (2002). An aliquot of the methanolic extract (500 µL) was mixed with 1.5 mL ethanol, 100 μL 10% AlCl_3_, 100 μL 1M potassium acetate, and 2.8 mL deionized water. The mixture was then incubated in the dark for 30 min. The absorbance was determined at a wavelength of 415 nm using a UV-Vis spectrophotometer. For the reference curve, the flavonoid rutin was used in a concentration range of 0.01 mg/mL at 0.5 mg/mL. The flavonoid content was expressed as milligram of rutin equivalent (RE) per gram of FW.

### 2.8 Data analysis

The experiments were performed with five plants per treatment and repeated three times, yielding similar results. Data were analyzed using one-way ANOVA followed by Tukey’s *post-hoc* test (*p*<0.05) using Statistica 7.0 software (Statsoft Inc.).

## 3 Results

### 3.1 VOCs from *B. bassiana* promote the growth of sorghum plants

**Figure 2:**
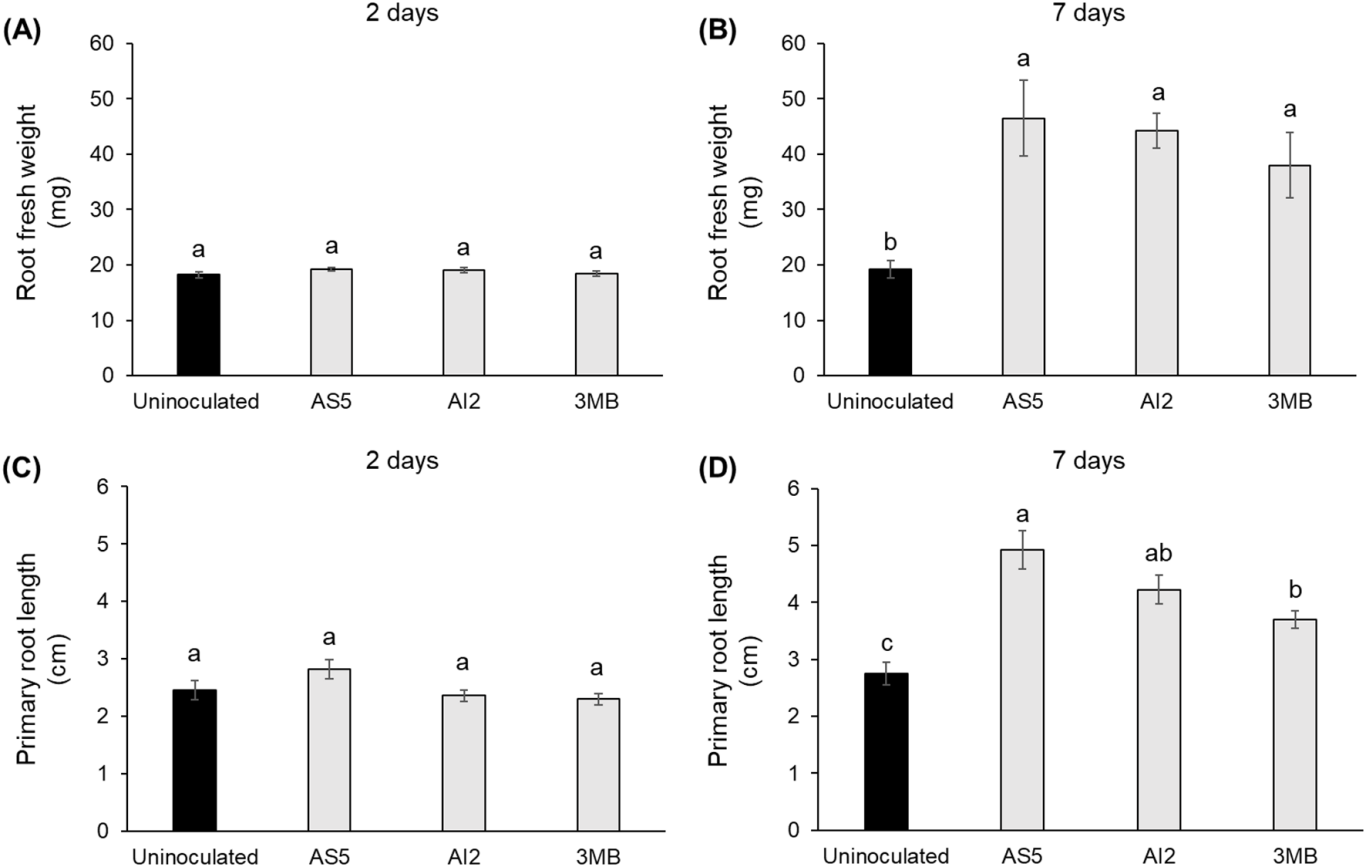
Effects of VOCs emitted by the strains AS5 and AI2 of *B*. *bassiana* and 3-methylbutanol on the root growth of sorghum plants. **(A, B)** Root fresh weight and **(C, D)** primary root length at 2 and 7d of control uninoculated plants and those plants exposed to the fungal VOCs and 3-methylbutanol exposure. Bars represent the mean ± standard error values (n = 5). Different letters are used to indicate means that differ significantly by one-way ANOVA and Tukey’s *post-hoc* test (*p* ≤ 0.05).

In this study, we conducted experiments to determine whether the VOCs emitted by a fungal entomopathogen promote plant growth. Therefore, the strains AS5 and AI2 of *B. bassiana* and sorghum plants were introduced into an *in vitro* system, in which they interacted only via VOCs emission (**Figure 1A**). Growth parameters such as shoot and root fresh weights, plant height, and primary root length were recorded after 2 and 7 d of interaction (**Figures 1** and **2**). In addition, the effect of 3MB, as the main compound emitted by both strains, was recorded. Plant growth promotion by fungal compounds was observed only at 7 d (**Figure 1B**). Statistically significant differences were observed in the aerial (**Figures 1D, F**) and root parts of the plant (**Figures 2B, D**). Interestingly, VOCs from AS5 had the strongest effect on plant growth. Notably, the VOCs emitted by AI2 and 3MB also stimulated the growth of foliage and roots compared with the control plants. Otherwise, IAA content in shoots was quantified by GC-MS to investigate whether the plant-promoting effect might be associated with an increase in auxin content. Results showed that plants treated with VOCs from AS5 increased the content of IAA two-fold in the foliar tissue. In contrast, IAA content in foliage from AI2 and 3MB treatments increased slightly, although they were not statistically different from the control (*p* ≤ 0.05) (**Table 1**).

**Table 1:** Content of phytohormones and phenolic compounds in sorghum leaves exposed to volatiles emitted by the strains AS5 and AI2 of *B. bassiana* and the standard 3-methylbutanol.

Compounds	Control	AS5	AI2	3MB
	(μg/g FW)			
Salicylic acid	0.02 ± 0.01 b	0.04 ± 0.01 a	0.03 ± 0.01 ab	0.04 ± 0.00 a
Jasmonic acid	0.05 ± 0.00 c	0.29 ± 0.04 b	0.22 ± 0.07 b	0.48 ± 0.04 a
Indole-3-acetic acid	0.15 ± 0.04 b	0.39 ± 0.06 a	0.25 ± 0.10 ab	0.19 ± 0.01 b
4-Coumaric acid	0.01 ± 0.00 c	0.03 ± 0.01 b	0.05 ± 0.00 b	0.07 ± 0.00 a
	(mg GAE/g FW)			
Total phenolics	83.04 ± 1.79 c	307.74 ± 18.07 a	156.70 ± 6.09 b	326.19 ± 20.42 a
	(mg RE/g FW)			
Total flavonoids	0.18 ± 0.01 c	1.49 ± 0.06 b	3.04 ± 0.11 a	3.25 ± 0.07 a
	(F/P ratio)			
Flavonoids/Phenolics	0.002	0.005	0.019	0.010

Phytohormones and phenols were analyzed by GC-MS and UV-Vis spectroscopy, respectively, after 7 d of the interaction. The total phenolic and flavonoid contents were expressed as mg gallic acid equivalents (GAE) and mg rutin equivalents (RE), respectively. Data are shown as means ± standard error (n = 5). Different letters indicate significant differences (P ≤ 0.05) among treatment, as determined by ANOVA and Tukey’s tests.

### 3.2 Reactive oxygen species and phenolic antioxidant production are induced in sorghum plants by fungal VOCs

**Figure 3:**
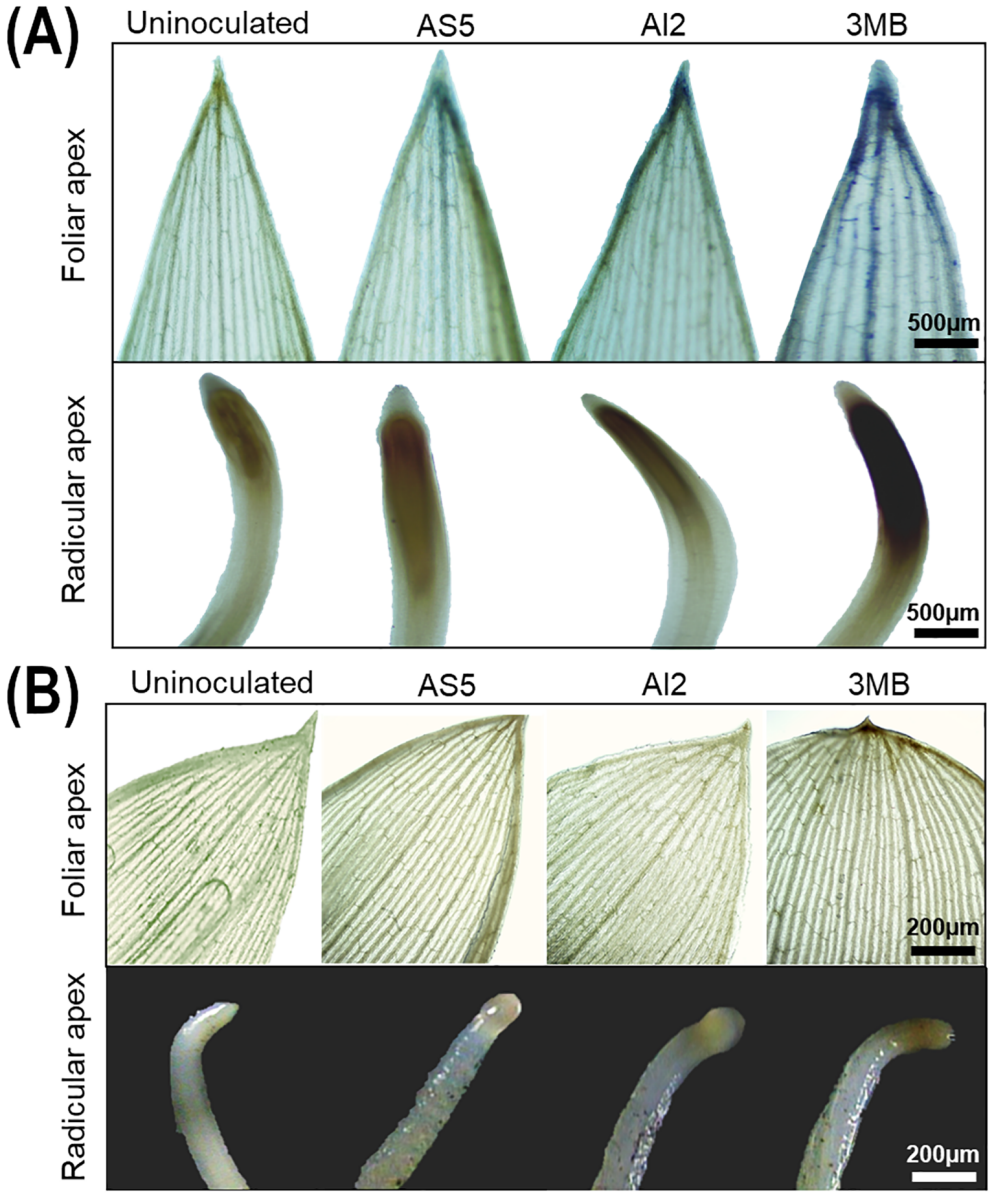
Representative photographs of leaves and roots from sorghum plants stained with **(A)** NBT to detect O_2_^•^¯ production, and **(B)** DAB for H_2_O_2_ in control uninoculated plants and after the exposure of VOCs emitted by AS5 and AI2 strains of *B*. *bassiana* and the compound 3-methylbutanol at 24h of the interaction.

ROS are important signals involved in plant physiology and responses to biotic and abiotic stresses (Sood, 2025). Therefore, we studied whether fungal VOCs stimulated ROS production after 24 h of plant-fungus interactions. NBT and DAB staining revealed the basal levels of O_2_^•^¯and H_2_O_2_, respectively, in the apical tissues of uninoculated plants (**Figures 3A, B**). O_2_^•^¯ is the first highly reactive form of oxygen formed in the chain of ROS, which leads to the formation of H_2_O_2_ (Haghpanah et al., 2025). When plants are exposed to VOCs emitted by AS5 and AI2, a visible hints on O_2_^•^¯and H_2_O_2_ production were observed in the apex, margin, and veins of leaves. Furthermore, the root tips appeared more stained compared to those of the control (**Figure 3**). Bioassays with 3MB suggest its role in inducing ROS production in leaves and roots, as higher accumulation of O_2_^•^¯and H_2_O_2_ was observed in apical tissues after 3MB exposure. Measurements of the total phenolic compounds and flavonoids in the leaves showed that, as a consequence of the interaction between plants and fungal compounds, the content of non-enzymatic antioxidants increased significantly (**Table 1**). Phenols from plants directly scavenge ROS, helping antioxidant enzymes control the sudden production of ROS (Khan et al., 2023). In the AS5 and 3MB treatments, the content of total phenolic compounds increased four-fold compared to the control. Indeed, the content of 4-coumaric acid, an abundant phenolic acid in sorghum grains (Xu et al., 2021), increases when plants are treated with fungal compounds. Consistently, flavonoids increased in relation to the control, by almost two-fold in AS5, and three-fold in AI2- and 3MB-treated plants. Notably, an increase in the flavonoid-to-phenol (F/P) ratio suggested that leaves exposed to fungal VOCs were richer in flavonoid content.

### 3.3 Effect of VOCs from *B. bassiana* on the expression of defense-related genes in sorghum

**Figure 4:**
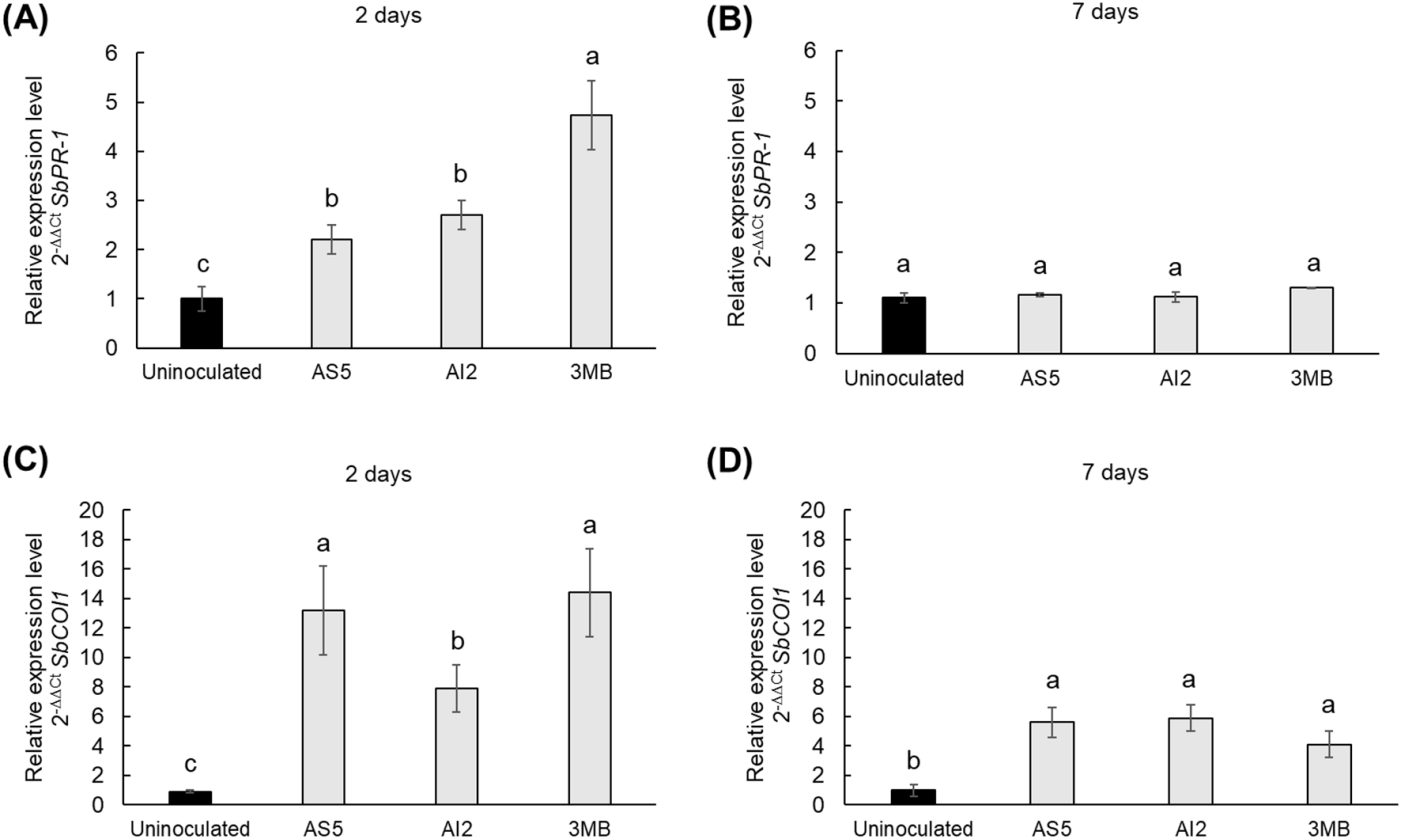
Effects of VOCs emitted by *B*. *bassiana* on the expression of *PR-1* and *COI1* defense genes in *S. bicolor*. Control uninoculated plants and those plants exposed to compounds emitted by AS5 and AI2 strains, and the standard 3-methylbutanol were harvested at 2 and 7d of the interaction to analyze the relative expression level of **(A, B)** pathogenesis-related protein 1 (*PR-1*) and **(C, D)** coronatine insensitive-1 (*COI1*) by qPCR. Bars represent the mean ± standard error values (n = 5). Different letters are used to indicate means that differ significantly by one-way ANOVA and Tukey’s *post-hoc* test (*p* ≤ 0.05).

Given the role of ROS in orchestrating local and systemic defense responses in plants, we analyzed the transcription of *SbCOI1*, implicated in the JA signaling pathway, and *SbPR-1*, as an inducible gene of the SA signaling pathway in sorghum plants (**Figure 4**). Necrotrophic phytopathogens and herbivores trigger JA-mediated defenses, whereas SA is frequently activated by hemi- and biotrophic pathogens (Monte, 2023). The results showed that fungal VOCs activated both, *SbPR-1* and *SbCOI1* at 2 d of the interaction, and the compound 3MB was an important elicitor of the up-regulation of the transcription of *SbPR-1* and *SbCOI1* genes (**Figures 4A, C**). The relative expression levels of *SbPR-1* in plants inoculated with AS5 and AI2 were similar, whereas *SbCOI1* had higher expression levels in plants exposed to VOCs from AS5 than in AI2-treated plants. Remarkably, *SbCOI1* expression was five-fold higher than *SbPR-1*. Moreover, after 7d of interaction, only *SbCOI1* was still overexpressed (**Figures 4B, D**). Consistently, the contents of SA and JA in foliar tissues increased after 7 d of fungal VOCs exposure (**Table 1**). Indeed, the JA content was four-fold higher than that of SA. Finally, the addition of 3MB induced the accumulation of both phytohormones in sorghum plants, confirming its role as a chemical cue during plant-fungus interactions.

## 4 Discussion

*Beauveria bassiana* is a beneficial fungus that has been widely studied for its ability to act as an insect pest suppressor, plant growth and defense promoter, plant disease antagonist, and plant inducer of resistance to biotic and abiotic stresses (Gonzáles-Mas et al., 2021; Ortiz-Urquiza, 2021; Gupta et al., 2022). Using field omics, molecular reprogramming has been observed in plants during the endophytic colonization. Transcriptomic, proteomic, and metabolomics tools have revealed that *B. bassiana* triggers plant photosynthesis, sulfur, iron, nitrogen, and phosphate metabolism-related genes, plant defense pathways, including SA and JA signaling pathways, and the production of secondary metabolites involved in the resistance against herbivorous insects or phytopathogen attackers (Raad et al., 2019; Proietti et al., 2023; Muola et al., 2023; Wang et al., 2023; Liu et al., 2025). Presently, we could add an extra attribute to *B. bassiana* that involves the activation of different biochemical traits in plants, such as ROS production, JA and SA signaling pathways, and the accumulation of phytohormones and phenols via fungal VOCs emission.

Endophytic interactions between *B. bassiana* and sorghum plants have been previously reported (Tefera and Vidal, 2009; Reddy et al., 2009). *Beauveria bassiana* was able to endophytically colonize the entire plant regardless of the inoculation method, although colonization was greater in the aerial part of the plant than in the roots. The benefits of fungal colonization in sorghum are diverse and include promoting plant growth, nutrient uptake and assimilation, and protection against pathogens and herbivorous insects (Mantzoukas et al., 2014; Raya-Díaz et al., 2017). These benefits have been observed in other crops, such as cucumber (*Cucumis sativus*), tomato (*Solanum lycopersicum*), melon (*Cucumis melo*), and tea (*Camellia sinensis*), among others (Tall and Meyling, 2018; Shaalan et al., 2021; González-Mas et al., 2023; Russo et al., 2023; Liu et al., 2025; Xie et al., 2025). However, to the best of our knowledge, the role of VOCs emitted by *B. bassiana* as chemical cues for its interaction with plants or as a plant growth promoter has yet to be elucidated.

The importance of volatiles from entomopathogenic fungi, such as *Beauveria*, *Metarhizium*, and *Isaria* has been demonstrated in insect pathogenesis; these fungi constitutively produce these compounds and act as elicitors of immune and behavioral responses in insects (Hussain et al., 2009; Zhang et al., 2024). However, the physiological effects of these fungal compounds in plants remain unclear. Remarkably, we observed that the VOCs emitted by *B. bassiana* promoted plant growth and defense. At 7 d of interaction, plants inoculated with AS5 and AI2 showed a higher shoot and root fresh weights, heights, and primary root lengths, which may have resulted in increased IAA content. Previously, it was reported that colonization of *Nicotiana benthamiana* by *B. bassiana* resulted in auxin accumulation and increased expression of biosynthetic pathway genes (Liu et al., 2025). Therefore, it is tempting to speculate that the pool of VOCs in *B. bassiana* promotes plant growth via an auxin-dependent mechanism. Notably, plants respond to VOCs emitted by microbes through highly conserved regulatory mechanisms. Evidence provided using transgenic *Arabidopsis* plants interacting with rizosphere and non-rizosphere bacteria and fungi, including pathogens show a modulation in hormone synthesis and signaling (e.g., auxin, ethylene, jasmonic acid, and cytokinin) (Ryu et al., 2003; Gutiérrez-Luna et al., 2010; Sánchez-López et al., 2016; Garnica-Vergara et al., 2016; Raya-González et al., 2017). In particular, pure compounds have been assayed for efficacy in promoting plant growth across varying doses. Furthermore, signal transduction pathways have revealed the specificity of the plant responses; for example, C16-dimethylamine (1-5 μM) from *A. agilis* UMCV2 regulates root morphogenesis via jasmonic acid signaling and the root responses to 6-pentyl-2*H*-pyran-6-one (50-200 μM) emitted by *T. atroviride* IMI206040 involve auxin transport and signaling and the ethylene-response modulator *ETHYLENE INSENSITIVE2* (*EIN2*) (Garnica-Vergara et al., 2016; Raya-González et al., 2017). These findings may indicate that microbes have evolved multifaceted metabolic capabilities to interact with their host plants. In this study, bioassays performed with 3MB emitted by *B. bassiana* showed activity as a plant growth promoter. However, further studies are needed to identify the sets of genes that are affected or unaffected in plants by the exposure to the fungal alcohol.

Other data presented in this work show that AS5 had a greater effect on plant growth promotion than AI2. The ability of soil microbes to produce VOCs varies according to their lifestyle and habitat, because they play a significant role in their adaptation to the environment (Bills and Gloer, 2016). The VOC profiles differed between AS5 and AI2, which might be attributable to the environmental sources from which they were isolated; AS5 was isolated from soil and AI2 from a mycosed insect cadaver. Furthermore, spores from AI2 showed higher virulence against the insect herbivorous *S. frugiperda* than those from AS5, and VOCs from AI2 had a greater effect on oviposition and feeding behaviors in the adults and larvae of *S. frugiperda* than those from AS5 (Ramírez-Ordorica et al., 2022; 2024). This evidence shows that VOCs released by entomopathogenic fungi are important mediators of “communication” between insects and plants. However, the mechanisms underlying the specificity of the response in each organism remain unknown.

Plants establish different relationships with soil microbes depending on their VOC profiles, ranging from beneficial to pathogenic or commensal (Hernández-Calderón et al., 2018). Undoubtedly, plants respond to microbial VOCs by activating defense responses, and, according to VOC fingerprints, they may act as activators or suppressors of these responses (Erb, 2018; Fincheira et al., 2021). In the case of VOCs from AS5 and AI2, NBT and DAB staining revealed an active production of O_2_^•^¯and H_2_O_2_ in sorghum plants at 24 h of the interaction. In addition, fungal VOCs and 3MB activate SA- and JA-dependent defense responses in sorghum plants. Similarly, during root colonization, some fungal-derived proteins, such as those involved in chitin signaling and fungal resistance, act as elicitors of these biochemical responses in plants (Hanan et al., 2020; Hu and Bidochka, 2021; Raad et al., 2019; Nazir et al., 2020). Furthermore, our data showed that exposure to fungal VOCs stimulates the synthesis of 4-coumaric acid, a compound previously identified as putative in sorghum plants by Ramírez-Ordorica et al. (2024). Consistently, phenolic compounds and flavonoids were found to be increased, probably as protective substances to counteract oxidative stress caused by changes in ROS homeostasis. Previous studies have highlighted the ability of *B. bassiana* to trigger the synthesis of phenolic compounds during root colonization and to protect plants against phytopathogens and herbivorous insects (Shaalan et al., 2021; Muola et al., 2023; Tyurin et al., 2023; Wang et al., 2023). Therefore, the pool of VOCs emitted by *B. bassiana* may promote plant growth and activate phenylpropanoid metabolism, which represents the core link in the biosynthesis of phenolic compounds; the increased content of the phenylpropanoid compound 4-coumaric acid observed in treated plants could consequently lead to an increase in flavonoid content (Xu and Wang, 2025).

Previously, Hernández-Calderón et al., 2018 reported statistical differences in expression of *SbPR-1* and *SbCOI1* when sorghum plants interacted for 8 d with beneficial (*A. agilis* UMCV2, *B. methylotrophicus* M4-96, and *Sinorhizobium meliloti* 1021), pathogenic (*Pseudomonas aeruginosa* PAO1), and commensal (*Bacillus* sp. L2-64) bacteria. The induction of *SbPR-1* and *SbCOI1* was higher in plants exposed to VOCs emitted by UMCV2 and 1021 strains, suggesting that both bacterial species may induce both SAR and ISR immune mechanisms. Otherwise, pathogenic bacteria strongly induced *SbPR-1*, whereas commensal bacteria did not upregulate transcription of *SbPR-1* and *SbCOI1*. These results revealed that plants differentially respond to microbial volatile signals. Exposure of sorghum plants for two days to fungal VOCs increased the relative expression levels of *SbPR-1* and *SbCOI1*. At a later stage of the interaction (7 d), only the expression of *SbCOI1* remained active, and the contents of both SA and JA were higher than in uninoculated plants. Studies across different plant species inoculated with *B. bassiana* reported that during fungal colonization, plants activate both the SA- and JA-pathways; when symbiosis is well established, the SA-pathway is repressed, and JA-responsive genes are upregulated, accompanied by an increase in JA content. Therefore, the interaction between *Beauveria* and plants seems to be complex, because both the SA and JA signaling pathways are activated by the fungus at an early stage of the interaction (2 d), which may lead to an integrated immune response during colonization (Raad et al., 2019; Proietti et al., 2023; Liu et al., 2025). Once the symbiosis is established, the ISR immune mechanism remains activated by the entomopathogenic fungus.

Consistently, 3MB stimulated the accumulation of SA, JA, and phenolic compounds in sorghum plants and up-regulated the transcription of *SbPR-1* and *COI1* genes suggesting that alcohol plays an important role in triggering ISR (induced systemic resistance) and SAR (systemic acquired resistance) immune responses in plants. 3MB is a by-product derived from fermentation of the amino acid leucine (Ravasio et al., 2014), and microorganisms with different lifestyles such as *B. bassiana, Saccharomyces cerevisiae*, *Metschnikowia reukaufii*, *Fusarium oxysporum*, *Trichoderma atroviride*, and *Bacillus* sp., among others, are able to produce this compound; therefore 3MB has been studied from different biotechnological perspectives because this compound shows activity in plants, insects, and microorganisms, either alone or in a complex mixture of compounds; moreover, the effect of 3MB may vary according to the dose, time of exposure, and application method (Wang et al., 2022; Mulero-Aparicio et al., 2019; Plaszkó et al., 2020; Hou et al., 2021; Ramírez-Ordorica et al., 2022; Lochmann et al., 2024; Bitas et al., 2015; Ma et al., 2025).

**Figure 5:**
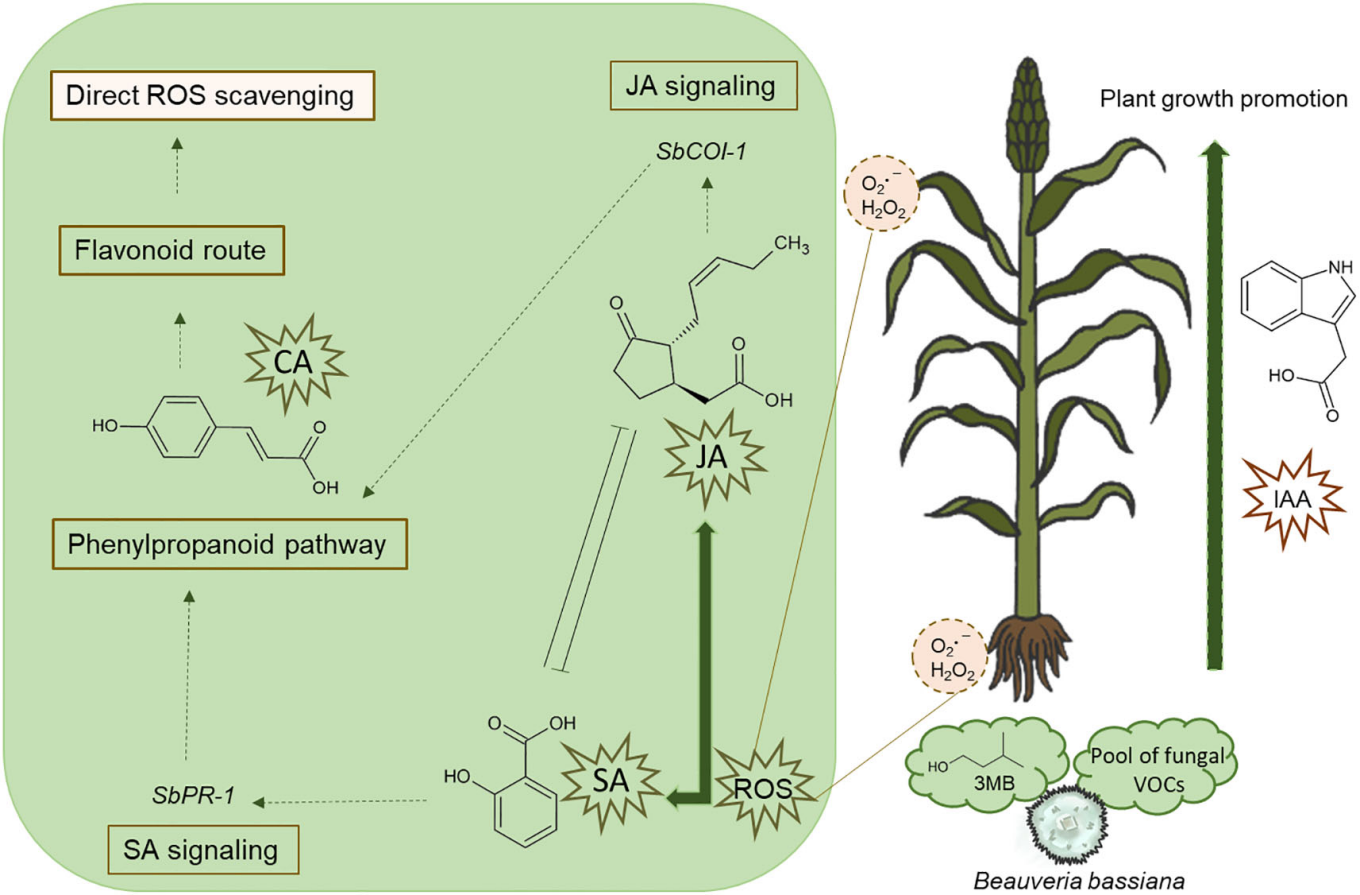
A simplified model of the plant response during the perception of the pool of VOCs emitted by the entomopathogenic fungus *B*. *bassiana* and the standard 3-methylbutanol (3MB). The plant host recognition of fungal VOCs triggers early signaling events such as oxidative burst with the subsequent activation of plant growth promotion, and SA and JA-mediated defenses; which were evident in (1) the accumulation of the phytohormones, indole-3-acetic acid (IAA), salicylic acid (SA), and jasmonic acid (JA), (2) the activation of plant defense genes *PR1*, as an inducible gene of the SA signaling pathway, and *COI1* implicated in the JA signaling pathway, and (3) the redox regulation with the accumulation of 4-coumaric acid (CA), a precursor of flavonoids with free radical scavenging and antioxidant activities.

The effects of volatile microbial compound on plant growth and defense have been studied for two decades. During this time we have highlighted the following: (1) soil microbes produce a wide array of VOCs, and VOCs profiles are species/strain distinctive; notably (2) the plants are able to detect VOCs emitted by a plethora of microorganisms and the biochemical response depends on the microbial emission source (beneficial vs. pathogenic), thus, the compounds can be recognized as safe, promoting plant growth, or not; (3) the promotion of plant growth involves highly conserved molecular mechanisms; (4) the identification of which compound(s) are responsible for such effects in plants is complicated because these compounds are volatile at room temperature, which makes their study, either alone or in combination, difficult; however (5) microbial VOCs are widely recognized as plant growth promoters and/or activators of plant defenses, alleviating biotic and abiotic stresses; therefore they are alternative players for organic agriculture. Our data strengthen and expand knowledge of the biochemical response of plants to microbial VOCs by showing that plant growth-promoting effects extend to phylogenetically distant microbial species, including entomopathogenic fungi. Furthermore, the establishment of a symbiotic relationship with an entomopathogenic fungus leads to the combined activation of SA and JA signaling. Thus, we hypothesize that volatiles from *B. bassiana* stimulate ROS production, which is linked to the growth of plants and defense; consequently, the content of phytohormones such as IAA, SA, and JA increases in the plant, modulating SA- and JA- signaling pathways, and as the interaction progresses over time, the plant may recognize the fungus as beneficial for repressing the SA pathway. Furthermore, to overcome oxidative damage, fungal VOCs activate the phenylpropanoid pathway, increasing the phenolic compound content required for ROS scavenging (**Figure 5**). In addition, 3MB may be part of the “chemical signal” that plants recognize to activate plant growth and defense programs. In conclusion, VOCs emitted by *B. bassiana* and 3MB elicit growth and defense responses in sorghum plants through the activation of JA and SA signaling pathways, and the accumulation of phenols and auxins; therefore, 3MB may function as a cross-kingdom signal, mediating complex interactions with plants. These findings provide new insights into how plants respond to the VOCs emitted by an entomopathogenic fungus that naturally colonizes plants endophytically. Finally, we consider that these results may offer important guidance for subsequent research and management of plant-*Beauveria* interactions for sustainable agriculture.

## Data Availability

The original contributions presented in the study are publicly available. This data can be found here: Zenodo, https://zenodo.org/records/18247794, https://zenodo.org/records/18248209.
