# Supplementary material for: Volatiles emitted by the entomopathogenic fungus Beauveria bassiana elicit growth and defense in sorghum plants
Source: Front Fungal Biol. 2026 Jan 19;6:1725103. doi: 10.3389/ffunb.2025.1725103 (PMC12862943; doi:10.3389/ffunb.2025.1725103)
Supplement: Supplementary file 1 [file DataSheet1.docx]

Supplementary Material

# Supplementary Figures and Tables

## Supplementary Figures


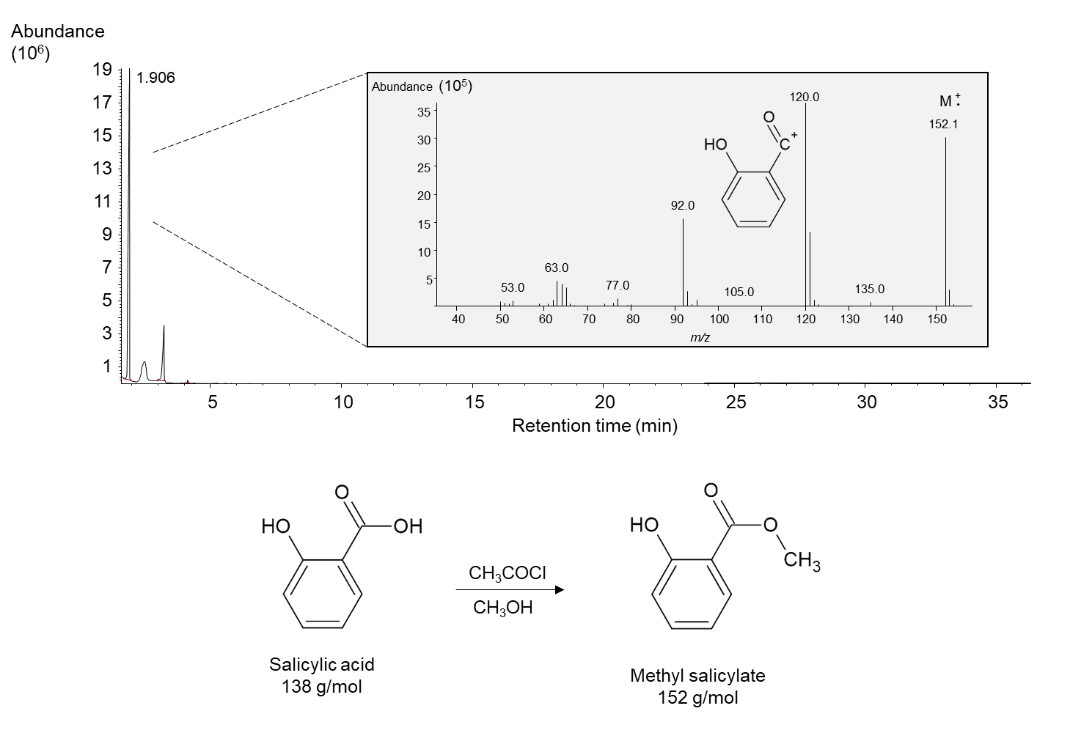


**Supplementary Figure 1.** Analysis of salicylic acid by gas chromatography and mass spectrometry (GC-MS). The phytohormone was derivatized with acetyl chloride in methanol to form methyl salicylate. The esterified compound eluted at 1.906 min. The gray rectangle shows the mass spectrum of the compound, and the ions 120 and 152 *m/z* were used to perform a selective ion monitoring (SIM) method for sample analysis.


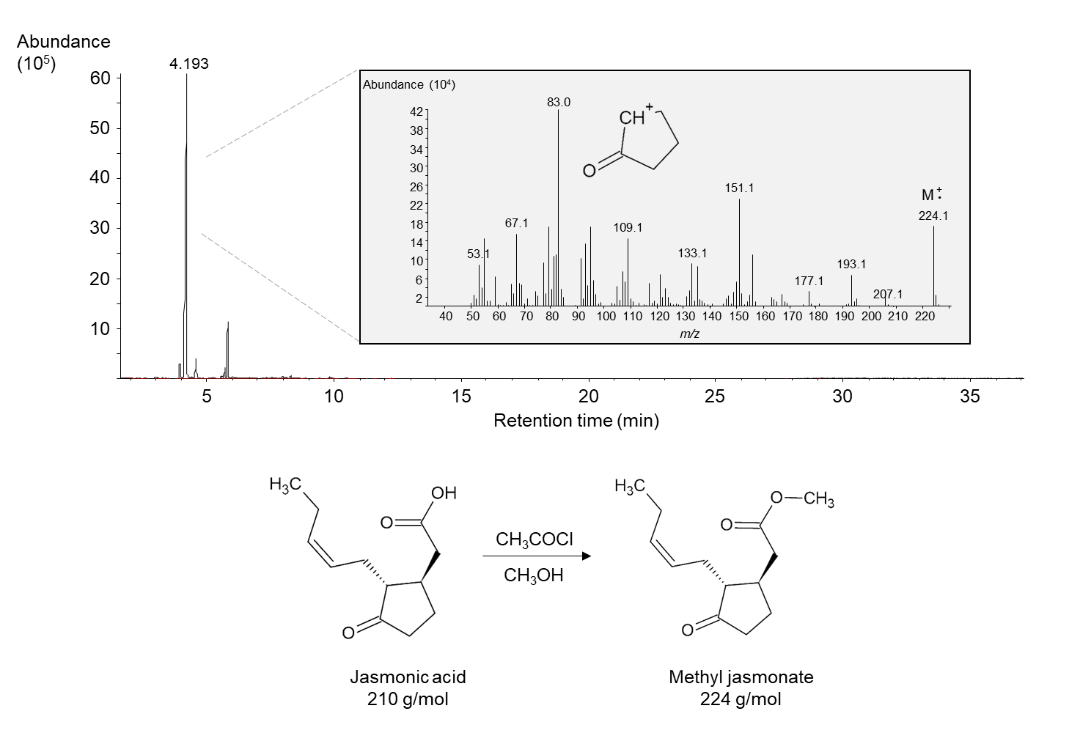


**Supplementary Figure 2.** Analysis of jasmonic acid by gas chromatography and mass spectrometry (GC-MS). The phytohormone was derivatized with acetyl chloride in methanol to form methyl jasmonate. The esterified compound eluted at 4.193 min. The gray rectangle shows the mass spectrum of the compound, and the ions 83 and 224 *m/z* were used to perform a selective ion monitoring (SIM) method for sample analysis.


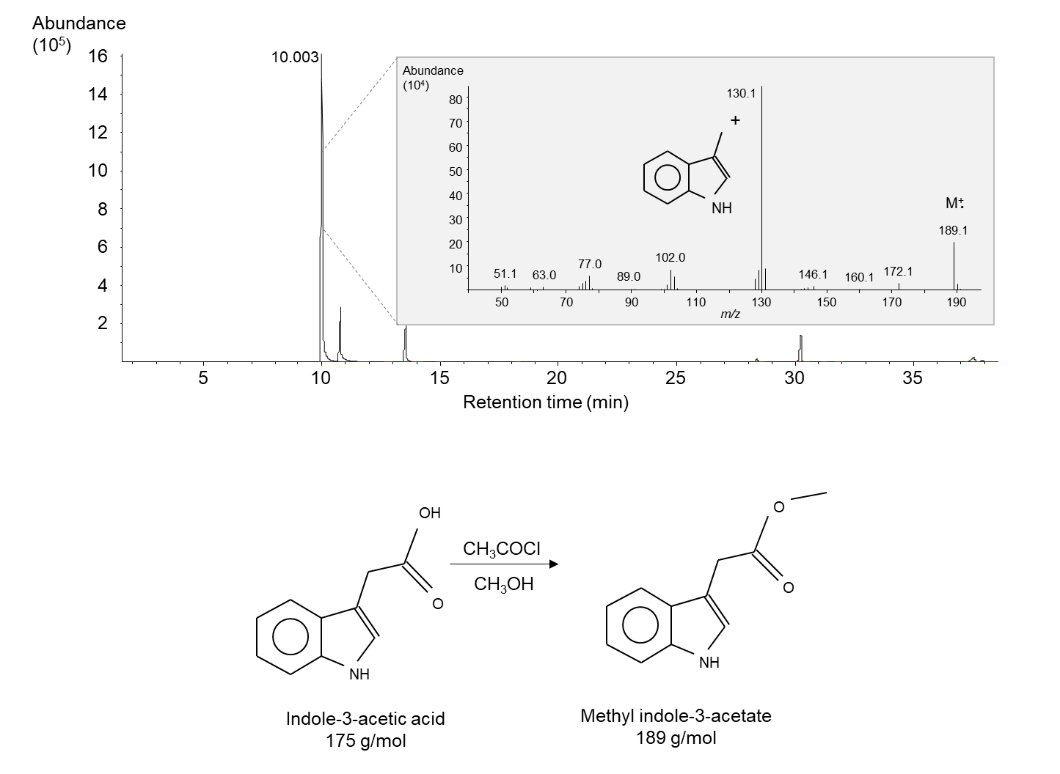


**Supplementary Figure 3.** Analysis of indole-3-acetic acid by gas chromatography and mass spectrometry (GC-MS). The phytohormone was derivatized with acetyl chloride in methanol to form methyl indole-3-acetate. The esterified compound eluted at 10.003 min. The gray rectangle shows the mass spectrum of the compound, and the ions 130 and 189 *m/z* were used to perform a selective ion monitoring (SIM) method for sample analysis.


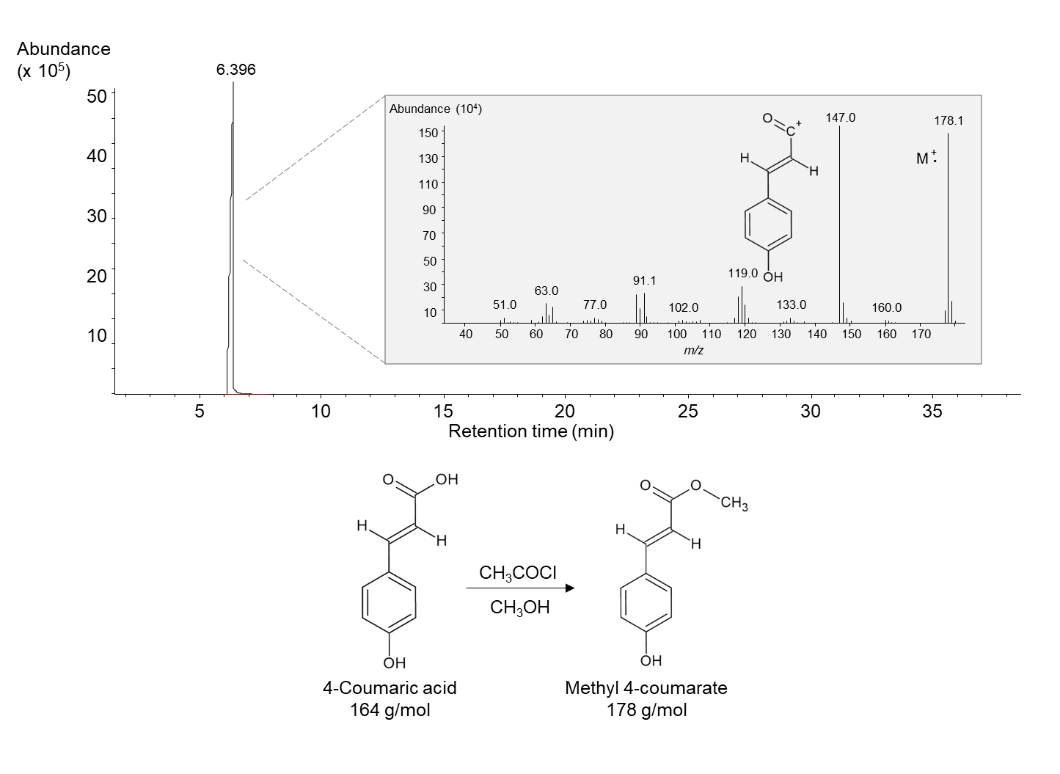


**Supplementary Figure 4.** Analysis of 4-coumaric acid by gas chromatography and mass spectrometry (GC-MS). The phenolic compound was derivatized with acetyl chloride in methanol to form methyl 4-coumarate. The esterified compound eluted at 6.396 min. The gray rectangle shows the mass spectrum of the compound, and the ions 147 and 178 *m/z* were used to perform a selective ion monitoring (SIM) method for sample analysis.

## Supplementary Table

| Supplementary Table S1. Characteristics of the primer pairs used to amplify defense and control genes in sorghum plants by RT-PCR (Hernández-Calderón et al., 2018). | | | | |  |
| --- | --- | --- | --- | --- | --- |
| **Accession number**  **NCBI/SbGBD** | Functional annotation | Code | Primer pair | Melting  Temperature (°C) | |
| **XM_002436360.1/Sb10g001940.1** | Pathogenesis-related protein 1 | *PRP1* | F.TCATCTGCAACTACGAGCCC  R. ACGTGTCTGTGTAAGCCGTC | 63 | |
| **SbXM_002456604.1/ Sb03g040150.1** | Coronatine-insensitive protein 1 | *COI1* | F.GGACTTGCAGTACACTTGCC  R.ACCAGTAGGTTGGGGCATTT | 63 | |
| **X79378.1** | Actin | *ACT* | F. TCTGGCATCACACCTTCTAC  R. GTACGACCGCTGGCATAG | 63 | |
